# In Vivo Targeted Reprogramming of Cardiac Fibroblasts for Heart Regeneration: Advances and Therapeutic Potential

**DOI:** 10.3390/bioengineering12090940

**Published:** 2025-08-30

**Authors:** Waqas Ahmad, Suchandrima Dutta, Xingyu He, Sophie Chen, Muhammad Zubair Saleem, Yigang Wang, Jialiang Liang

**Affiliations:** 1 Department of Pathology and Laboratory Medicine, College of Medicine, University of Cincinnati, Cincinnati, OH 45267, USA; ahmadws@ucmail.uc.edu (W.A.);; 2 Department of Internal Medicine, College of Medicine, University of Cincinnati, Cincinnati, OH 45267, USA; duttasm@mail.uc.edu; 3 Department of Pharmacology, Physiology and Neurobiology, College of Medicine, University of Cincinnati, Cincinnati, OH 45267, USA

**Keywords:** cardiac reprogramming, myocardial infarction, heart failure, cardiac fibroblasts, cardiomyocytes, regeneration, fibrosis, gene delivery

## Abstract

Myocardial infarction-induced cardiovascular diseases remain a leading cause of mortality worldwide. Excessive post-infarct fibrosis contributes to adverse cardiac remodeling and the progression to heart failure. In vivo reprogramming strategies offer a promising avenue for heart regeneration by directly converting resident fibroblasts into cardiomyocytes through enforced expression of cardiogenic genes. This approach circumvents the need for invasive biopsies, cell expansion, induction of pluripotency, or autologous transplantation. Despite these advantages, key challenges persist, including low reprogramming efficiency and limited cellular targeting specificity. A critical factor for effective anti-fibrotic therapy is the precise and efficient delivery of reprogramming effectors specifically to fibrotic fibroblasts, while minimizing off-target effects on non-fibroblast cardiac cells and fibroblasts in non-cardiac tissues. In this review, we discuss the cellular and molecular mechanisms underlying in vivo cardiac reprogramming, with a focus on fibroblast heterogeneity, key transcriptional drivers, and relevant intercellular interactions. We also examine current advances in fibroblast-specific delivery systems employing both viral and non-viral vectors for the administration of lineage-reprogramming factors such as cDNA overexpressions or microRNAs. Finally, we underscore innovative strategies that hold promise for enhancing the precision and efficacy of cellular reprogramming, ultimately fostering translational development and paving the way for rigorous preclinical assessment.

## 1. Introduction

Myocardial infarction (MI), hypertension, cardiomyopathies, and ischemia or reperfusion injury are the widespread medical conditions that result in heart failure [1]. These pathologies cause the death of numerous functional cardiomyocytes (CMs) and activate pathological remodeling, resulting in the replacement of contractile myocardium with fibrous tissue [2]. Although there have been impressive developments in pharmacology and device-based therapies for the treatment of heart failure, available therapeutic options can only handle symptoms and slow down the disease progression [3]. Heart transplantation is still the only option for patients with advanced heart failure who have not responded to other treatments [4]. However, it also has some limitations, for instance, donor availability, the need for immunosuppression, risk of immune rejection, and indicating the pressing demand for new treatment options [5].

Regenerative medicine has surfaced as a symbol of hope for confronting heart failure fundamentally by regaining the heart’s lost functionality instead of just mitigating symptoms [6]. Among the different approaches of regenerative medicine, stem cell-based treatment has drawn significant attention [7]. These involve the transplantation of cardiovascular cells derived from induced pluripotent stem cells (iPSCs) [8], mesenchymal stem cells [9], or other progenitor cells to regenerate necrotic myocardium [10]. Nonetheless, despite potentially successful preliminary clinical results, stem cell-based treatment faces major challenges, e.g., post-transplantation arrhythmia [11], immune rejection [12], oncogenic potential [13], and the hurdle of bulk differentiation into functional CMs [14]. Additionally, the harsh microenvironment in the compromised heart also impedes the therapeutic efficacy by causing rejection and the death of the transplanted cells [15].

Considering these obstacles related to stem cell treatment, direct cellular reprogramming has emerged as a radical substitute. Driven by iPSCs studies, cell fate alteration has resulted in the development of induced cardiomyocytes (iCMs) via delivering specific transcription factors into somatic cells, such as skin or cardiac fibroblasts (CFs) [16,17,18]. This direct reprogramming has been further refined to target resident fibroblasts in the heart. This induced conversion into CM-like cells replenishes the injured heart in situ [19,20]. This direct reprogramming may avoid the potential risk of tumorigenesis associated with pluripotent stem cells. Additionally, the reprogramming of autologous cells reduces the chance of immune rejection and fibrosis because scar tissue transforms into functional myocardium [21,22,23]. These advantages demonstrate direct cell fate conversion as a more effective and morally acceptable approach for cardiac regeneration.

Direct cardiac reprogramming can be pursued through either in vitro or in vivo strategies. In vitro reprogramming involves converting fibroblasts into iCMs in culture dishes through genetic modifications [24], offering a controlled platform to study lineage conversion. Although transplantation of iCMs into injured myocardium demonstrates promising functional recovery [16], the translational potential remains limited. Both iCMs and iPSC-derived CMs display comparable molecular signatures [25]; however, whether iCMs represent a superior therapeutic source for MI is unresolved. Importantly, iCM transplantation faces inherent challenges, such as poor survival and limited integration, restricting its therapeutic use [26]. Consequently, in vitro iCM approaches are more valuable for mechanistic studies than for direct clinical application. In contrast, in vivo reprogramming offers a more streamlined and clinically applicable alternative [27,28]. As it reprograms the fibroblasts residing inside the heart, it thus bypasses the necessity of the external cells’ replication and transplantation [29]. It simplifies the therapeutic process as well as utilizes the inherent CFs, thus nurturing cell survival and functional recovery [30]. Therefore, this review highlights recent advances in in vivo cardiac reprogramming.

The challenge is the efficient and definite delivery of the reprogramming factors to the targeted cells for successful in vivo reprogramming. In particular, fibrotic regions characterized by a mature, densely cross-linked scar matrix pose an additional barrier to efficiently targeting CFs, as the compact extracellular architecture restricts the penetration and distribution of regenerative therapeutic agents. Therefore, formulating optimized cocktails of the reprogramming elements and effective delivery methods plays a crucial role in translating this conception. These approaches ensure the targeted delivery of the reprogramming factors, making them more accessible for cellular uptake and preventing unintended effects. Hence, a systematic review is important to provide clarity and direction for future research. This review offers a comprehensive overview of the latest advancements in accurate and efficient in vivo delivery of the reprogramming factors for the treatment of heart diseases. It provides a detailed discussion of the key processes regulating cardiac reprogramming in vivo, including reprogramming factors, cellular targets, and the interactive relationship of the tissue environment. Moreover, we analyze the benefits and limitations of the wide range of genes and drug delivery systems. By combining the available findings, our objective is to highlight progress in the field and put forward systematic approaches for the future development of in vivo cardiac reprogramming.

## 2. Biological Basis of In Vivo Cardiac Reprogramming

Recent investigations have shown that lineage transdifferentiation can be achieved in vivo by introducing specific reprogramming agents [31,32], presenting a possible approach for heart regeneration. Nevertheless, the exact processes governing in vivo cardiac reprogramming are inadequately comprehended owing to the intricate tissue environment and the heterogeneous cellular composition of the heart. Extracellular signaling, intracellular epigenetic changes, and intercellular cell–cell interactions presumably significantly influence the efficiency and fidelity of reprogramming. A comprehensive understanding of the cellular mechanisms regulating in vivo cardiac reprogramming is crucial for enhancing this methodology.

### 2.1. Impacts of Fibroblast Origin, Differentiation State or Age

As the abundant non-CM cells in the heart, CFs have served as the preferred starting point for in vivo cardiac reprogramming [31]. Recent lineage-tracing studies such as Tcf21 (transcription factor 21)-iCre and Fsp1 (fibroblast-specific protein-1)-Cre have confirmed the successful conversion of a subset of CFs into iCMs, demonstrating their integration into the myocardium and contributions to ventricular contractility [19,20,33]. The origin, differentiation state, or age of fibroblasts may significantly affect both the efficiency of conversion and the quality of the resulting iCMs.

Importantly, CFs play a key role in maintaining the structural integrity of the heart [34]. Following cardiac injury, these cells become activated and secrete extracellular matrix (ECM) proteins, leading to fibrosis and pathological remodeling [34]. Single-cell RNA sequencing across various mouse models of heart disease and human cardiac tissue has revealed the heterogeneous nature of CF populations [35]. Single-cell analysis of transgenic models of in vivo cardiac reprogramming has identified both quiescent and activated fibroblast subtypes, suggesting that reprogramming factors may promote a shift toward an anti-fibrotic state [36]. However, it remains largely unclear whether specific fibroblast subtypes differ in their reprogramming potential, including conversion efficiency, iCM subtype specification, or functional maturation. Developing novel genetic lineage-tracing mouse models could help identify fibroblast subpopulations with distinct molecular signatures that are more amenable to efficient in vivo cardiac reprogramming. Interestingly, cardiogenic transcription factors such as Tbx20 (T-box transcription factor 20), Gata4 (GATA-binding protein 4), and Hand2 (heart and neural crest derivatives expressed 2) are enriched in CFs and are involved in both cardiac homeostasis and disease [37]. Although their basal expression may be insufficient to initiate reprogramming, these factors could serve as molecular markers of subpopulations more responsive to transdifferentiation, warranting further investigation.

Moreover, the differentiation status of CFs can markedly influence the outcomes of in vivo reprogramming. The dynamic trajectory of CF development and differentiation can be characterized through single-cell RNA sequencing and lineage-tracing techniques. Following ischemic or non-ischemic injury, quiescent CFs rapidly proliferate and transition into αSMA (alpha smooth muscle actin)- or Postn (periostin)-expressing myofibroblasts, which represent the predominant ECM-producing population in the heart [35]. Notably, while Postn expression is not exclusive to activated fibroblasts during development or in other tissues, in adult stressed cardiac ventricles it is specifically expressed by activated fibroblasts [38]. The potential to reprogram myofibroblasts into iCMs has been demonstrated using Postn-lineage tracing [39], although the efficiency is lower compared to that observed in non-activated CFs. Consistently, αSMA-lineage tracing has revealed that the majority of iCMs generated from CFs in vitro do not undergo a transient myofibroblast stage [40]. These observations underscore the difficulty of reprogramming fully activated myofibroblasts into iCMs. Furthermore, during the progression of cardiac fibrosis, myofibroblasts may transition into matrifibrocytes with more mature ECM features [41], posing an even greater barrier to reprogramming and warranting further investigation.

Our previous studies, along with those of others, have demonstrated that aged cells exhibit significantly reduced transdifferentiation efficiency compared to their younger counterparts in response to pluripotency reprogramming factors [42,43]. In the context of cardiac reprogramming, neonatal CFs from day 1.5 mice display markedly higher efficiency in generating iCMs than adult CFs from 4-week-old mice [44], even under identical culture and induction conditions. A similar age-related decline in iCM reprogramming efficiency has also been observed in mouse skin fibroblasts [45]. Despite these findings, relatively few studies have explored the reprogramming potential of aged CFs in vivo. Given that heart failure primarily affects older individuals, targeting adult or aged CFs holds greater clinical relevance and potential therapeutic impact. However, age-dependent changes in the epigenetic landscape may pose substantial barriers to efficient iCM reprogramming. These challenges may be mitigated through metabolic interventions aimed at reducing mitochondrial oxidative stress, such as the activation of mitophagy or the modulation of dietary lipid intake [45]. Autophagy, a conserved catabolic process, has also been shown to be both induced during and essential for successful iCM reprogramming [46]. Moreover, key cardiogenic transcription factors, such as Gata4, are downregulated in aged CFs [47]. Therefore, age-related alterations in gene expression and epigenetic regulation must be taken into account when optimizing cardiac reprogramming protocols. Tailoring these approaches to accommodate age-specific cellular states will be critical for the development of effective and personalized cardiac regenerative strategies.

### 2.2. Molecular Drivers of In Vivo Cardiac Reprogramming

It is important to note that the field of in vivo cardiac reprogramming is still at a nascent stage. A growing body of work has identified key molecular drivers that enable CF-to-CM conversion. These drivers fall broadly into four categories, cardiac transcription factors, microRNAs, small molecules, and epigenetic modulators, each of which acts on distinct but complementary pathways to reprogram CFs into iCMs. A variety of in vivo reprogramming strategies, including vector-based delivery systems, gene interventions, and small molecules, have been explored primarily in rodent models through intramyocardial injections (Table 1). Among these, gene delivery remains the dominant approach due to the pioneering effects of cardiac transcription factors. While chemical methods for both in vitro and in vivo iCM reprogramming have demonstrated potential for pharmacological therapy [48,49,50], their efficacy remains limited.

Among the molecular drivers of cardiac reprogramming, transcription factors serve as the foundational components of most strategies. Their DNA-binding activity initiates the broad transcriptomic and epigenomic changes required for lineage conversion. While a single factor may activate specific genes, effective reprogramming generally relies on combinations of factors that refine DNA binding and promote chromatin accessibility, particularly at cardiac loci [68]. Together, these factors activate enhancer networks in fibroblasts, recapitulating patterns observed during embryonic cardiogenesis [69]. In parallel, microRNAs act as potent post-transcriptional regulators, reinforcing cardiac identity by modulating gene expression programs that control sarcomere assembly, calcium handling, and contractile function [53]. Small molecule combinations targeting signaling pathways relevant to heart development can be used to induce fibroblast reprogramming [50]. These studies reinforce that the principal mode of action of reprogramming factors is the activation of cardiac developmental pathways, which also constituted the foundation of initial factor-discovery strategies [70].

Despite recent advances, the overall efficiency of generating mature iCMs in vivo remains relatively low, especially when compared to the substantial loss of native CMs typically observed following MI. This discrepancy underscores a critical need for further optimization of in vivo reprogramming strategies. To date, various combinations of transcription factor cocktails, reprogramming modulators, and expression stoichiometries have been explored, but no universal or maximally effective protocol has emerged. Continued refinement of these parameters in animal models is essential to converge on a consensus reprogramming paradigm capable of robust and reproducible cardiac regeneration. For example, the incorporation of microRNAs (e.g., miR-1, miR-133, miR-208, and miR-499) modulating gene networks or small-molecule inhibitors (targeting Wnt or Tgfβ) alongside core cardiac transcription factors has shown promise in enhancing the efficiency and fidelity of in vivo cardiac reprogramming [56,57]. These combinatorial strategies hold potential not only for increasing iCM yield but also for improving functional integration and maturation within the injured myocardium.

Epigenetic modifiers also play a pivotal role in reprogramming the fate of CFs. For example, Bmi1 (a member of the Polycomb repressor complex 1) [44], Polycomb repressive complex 2 (H3K27 methyltransferases) [71], or Mll1 (H3K4 methyltransferase) [72] have been identified as epigenetic barriers to in vitro iCM generation. However, their role in in vivo cardiac reprogramming remains unvalidated. Notably, PHF7, a histone reader that recognizes di- and tri-methylated H3 lysine 4 (H3K4me2/3), can substitute for Gata4 to reprogram CFs into iCMs in combination with Mef2c (myocyte enhancer factor 2c) and Tbx5 [73]. This supports the idea that a combination of transcription factors and epigenetic modulators might enable efficient reprogramming with fewer cofactors. Although PHF7 overexpression alone does not induce fibroblast-to-iCM conversion in vitro, it can reprogram CFs and other non-myocytes into iCMs in vivo and restore cardiac function following MI [67]. Mechanistically, this is attributed to the maintenance of a partially active chromatin landscape at CTCF and Jun/Fos/AP-1 regulatory loci associated with endogenous cardiac transcription factors [67], highlighting the importance of activating native cardiac gene networks during iCM formation. Additionally, these findings underscore the importance of spatial cellular cross-talk, which is typically absent under in vitro conditions. The role of the microenvironment in cardiac reprogramming mechanisms is explored in the following section.

### 2.3. Cellular Mechanisms Governing In Vivo Cardiac Reprogramming

Transcriptomic and epigenetic mechanisms underlying lineage reprogramming in CFs, triggered by molecular drivers such as Gata4, Mef2c, and Tbx5, have been elucidated through high-throughput sequencing or single-cell transcriptomic profiling [68,69]. Transcriptomic changes encompass activation of cardiogenic regulatory networks, suppression of fibrogenic signaling, and RNA splicing modulation, whereas epigenetic mechanisms involve enhancer activation, histone modifications, and changes in chromatin accessibility following the binding of pioneer transcription factors to DNA at specific loci [31,74]. Notably, single-cell profiling of transcription factor-infected cells has identified distinct subpopulations emerging during reprogramming, including unreprogrammed fibroblasts, intermediate fibroblasts, pre-iCMs, and iCMs [75]. Cell trajectory modeling has further clarified how fibroblasts diverge toward either successful reprogramming or a refractory state [76], thereby yielding critical insights into the efficiency and temporal dynamics of the conversion process. Although most of these studies were performed in vitro, they offer valuable mechanistic insights into how reprogramming factors coordinately activate cardiac developmental pathways while suppressing the fibroblast gene program—mechanisms that may reflect those occurring in vivo during CF reprogramming.

Importantly, the development of transgenic mouse models expressing cardiac reprogramming factors, such as the MGTH combination (Mef2c, Gata4, Tbx5, and Hand2), offers a powerful platform to dissect the in vivo mechanisms of cardiac reprogramming [36,77]. The lineage-tracing via inducible Tcf21-iCre has shown that approximately 1–3% of CFs are reprogrammed into iCMs in models of heart failure induced by a high-fat diet or MI [36,77]. Notably, beyond direct lineage conversion, reprogramming factors, particularly Gata4, were found to attenuate CF activation and reduce fibrotic remodeling, thereby contributing to improved diastolic function [77]. These findings suggest that a subset of CFs overexpressing MGTH may not fully transdifferentiate into iCMs, yet still exert therapeutic effects by disrupting fibrogenic activity. This highlights the dual role of reprogramming factors: promoting cardiomyogenesis and modulating the pathological fibroblast response.

Given the complexity of the in vivo environment, other cellular bioprocesses may contribute to the enhanced overexpression of reprogramming factors. For instance, the introduction of seven reprogramming factors via modified mRNAs can efficiently convert a subset of CFs into non-beating or immature CM-like cells, potentially activating pro-angiogenic signaling and paracrine effects that support cardiac repair in infarcted tissue [64]. Notably, supplementation with angiogenic factors such as fibroblast growth factor and vascular endothelial growth factor has been shown to enhance cardiac reprogramming [78]. Therefore, potential cell–cell communication among non-CMs warrants further investigation to promote the formation and maturation of iCMs. Additionally, the immune microenvironment may influence CF reprogramming. After MI, macrophage-derived interferon-β can activate gene responses in CFs that inhibit iCM induction, whereas macrophage depletion or blockade of interferon receptor signaling enhances reprogramming efficiency in vivo [79]. However, it remains unclear whether the reprogrammed cells or targeted CFs exert reciprocal effects on the immune system. Importantly, innate immune cells comprise both pro-inflammatory and anti-inflammatory subtypes, each potentially playing distinct roles in cardiac regeneration and post-MI response [80]. Elucidating the specific immune influences on CF reprogramming under varying conditions will be essential for optimizing in vivo reprogramming strategies.

## 3. Fibroblast-Targeted Strategies for Cardiac Lineage Reprogramming

**Figure 1:**
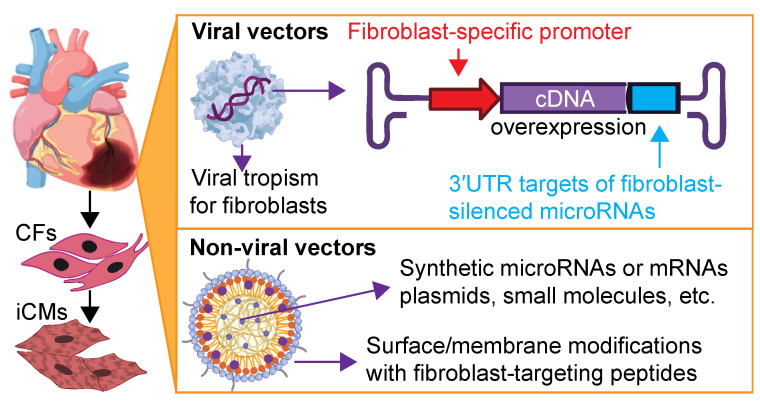
Recent advances in targeted delivery strategies for reprogramming factors to the infarcted heart. Viral or non-viral vectors have been developed to target CFs and deliver various reagents for in vivo cardiac reprogramming.

A key determinant of successful in vivo cardiac reprogramming lies in the precise and efficient delivery of reprogramming molecules, whether viral vectors, RNA species, or small molecules, specifically to CFs, while avoiding off-target effects on non-fibroblast populations such as CMs, endothelial cells, and fibroblasts in non-cardiac tissues. This consideration remains important for both systemic and localized delivery, as the circulatory system can redistribute therapeutic agents beyond the intended site [81]. Restricting the expression or cellular uptake of therapeutic agents to CFs may further promote anti-fibrotic outcomes by attenuating maladaptive fibrosis, thereby facilitating improved cardiac structure and function after MI. Central to these strategies is the identification and exploitation of molecular signatures or surface markers uniquely expressed by CFs (Figure 1), which can inform the development of diverse delivery platforms, from promoter-specific viral vectors to tailored nanoparticles.

### 3.1. Viral Vectors

Although retroviral and lentiviral vectors have been widely used in animal studies of in vivo cardiac reprogramming (Table 1), adeno-associated viruses (AAVs), which are non-integrative, less immunogenic, and already approved for gene therapy in certain genetic diseases [82,83], offer a more attractive platform for translational applications. Several AAV serotypes, such as AAV1, can efficiently transduce CFs for iCM reprogramming [62]. However, most AAVs engineered for gene delivery exhibit broad tropism, resulting in transgene expression across multiple cell types.

A common strategy for restricting gene expression to specific cell types involves the use of cell-type-specific promoters. Recently, several fibroblast-specific promoters, such as *Postn* and *Tcf21*, have been identified, and their core sequences have been utilized to construct AAV vectors for CF-restricted transgene expression [84,85]. Notably, AAVs carrying *Postn*-driven cardiac transcription factors have successfully induced in vivo reprogramming CFs into iCM in MI mouse models, leading to improved cardiac function [66]. However, the AAV genome size is limited to approximately 4.7 kb, including inverted terminal repeats and regulatory elements, posing significant constraints on packaging multiple transcription factors in a polycistronic format. This limitation can also compromise the cooperative activity of transcription factors needed to robustly activate the cardiogenic gene program.

Identifying a single, potent reprogramming factor could overcome this limitation and facilitate testing of *Postn*-driven AAVs. Notably, functional domains from reprogramming components such as MYOCD (Myocardin), ASCL1 (Achaete-Scute family BHLH transcription factor 1), and miR-133 can be packaged within a single AAV vector [86]. However, these constructs often fail to achieve the high expression levels needed for efficient cardiac reprogramming when driven by CF-specific promoters. To achieve CF-specific targeting while maintaining high expression, another strategy involves incorporating binding sites for CM-enriched microRNAs into the 3’ untranslated region (3’UTR) of the AAV transcript [86]. This allows robust expression under a constitutive promoter (e.g., CMV) in CFs, while suppressing expression in CMs. Nonetheless, this approach does not fully eliminate transgene expression in other non-myocyte populations, and therefore potential off-target effects remain a concern.

### 3.2. Non-Viral Vectors

As interest grows in safer and more adaptable methods, non-viral delivery systems such as polymeric materials and liposomes have emerged as promising alternatives to viral vectors for direct in vivo cardiac reprogramming. Pioneering studies employing peptide-modified cationic gold nanoparticles, loaded with Gata4, Mef2c, and Tbx5 plasmids and combined with polyethylenimine (PEI), successfully reprogrammed mouse embryonic fibroblasts (MEFs) and human skin fibroblasts into CM-like cells in vitro, and showed promise in reducing fibrotic scarring upon in vivo delivery [61]. Alternatively, cardiac-specific microRNAs have also been encapsulated in poly(lactic-co-glycolic acid) (PLGA)-PEI nanocarriers, demonstrating the potential to reprogram human fibroblasts into CM-like cells in vitro [87]. Furthermore, PEI-coated, nitrogen-enriched carbon dots have been identified as low-toxicity nanocarriers capable of delivering cardiac microRNAs to reprogram CFs in vitro and to reduce cardiac fibrosis with promoted angiogenesis when administered in vivo [88]. Nevertheless, whether these reported nanocomplexes can effectively target CFs in vivo and induce functional iCM formation remains unresolved. Further investigation employing stringent lineage-tracing methodologies is essential to confirm true cellular conversion.

To improve uptake efficiency and targeting specificity in cardiac cells, non-viral vector scaffolds are frequently functionalized with protein-affinity components (such as ligands, antibodies, or bioactive peptides). These modifications exploit receptor-mediated endocytosis as the dominant internalization mechanism, wherein nanoparticle–receptor complexes are actively trafficked into the intracellular compartment through vesicular pathways [89]. A notable example is a biomimetic delivery platform designed with a hybrid membrane shell (comprising artificial lipid membranes modified with tenascin-C-binding peptides and fused with neutrophil membrane proteins) and a mesoporous silica nanoparticle core carrying a cocktail of cardiac microRNAs [63]. This system has demonstrated efficient and specific targeting of CFs in vivo, leading to their transdifferentiation into induced iCMs, which may reduce myocardial fibrosis and enhance ventricular contractility [63]. However, the complexity of the system raises concerns about scalability, and the inherently poor biodegradability of silica nanoparticles further complicates clinical application.

Recently, extracellular vesicles (EVs) have gained attention as promising non-viral carriers for delivering cardiac reprogramming factors. EVs harvested from embryonic stem cells undergoing cardiomyogenic differentiation have been demonstrated to induce the direct reprogramming of MEFs into mature, functional iCMs in vitro [90]. Furthermore, intramyocardial delivery of these EVs in mouse models of myocardial infarction has been associated with reduced infarct size, decreased cardiac cell apoptosis, enhanced microvessel density, and improved cardiac performance [90]. Mechanistically, microRNAs such as miR-291 and miR-1 have been identified as key mediators in EV-facilitated cardiac reprogramming [90]. Nonetheless, the cellular specificity and mechanisms of action remain incompletely understood, particularly regarding whether CFs are the principal cellular targets and the extent to which the lineage transdifferentiation contributes to functional recovery. Despite their natural therapeutic potential, clinical translation of EV-based therapies is hampered by production scalability issues, heterogeneity, batch variability, and limited control over cargo composition [91]. Progress in strategies of in vivo cardiac reprogramming may be accelerated by designing synthetic vesicles that replicate the essential biochemical and biophysical properties of natural EVs while incorporating features for CF-specific targeting [92,93].

## 4. Challenges and Future Perspectives

The ultimate goal of in vivo cardiac reprogramming is to develop an effective regenerative therapy for heart failure patients. Bringing this strategy closer to clinical application requires the systematic identification and optimization of reprogramming factor(s) that perform effectively in human cellular contexts, followed by thorough validation in physiologically relevant animal models to assess safety and efficacy. While numerous factor cocktails (ranging from transcription factors to microRNAs and small molecules) have been explored, a universally effective and reproducible regimen remains elusive [94]. This is largely due to the intrinsic heterogeneity of the targeted starting cells-CFs that can vary considerably across species, developmental stages, and disease conditions [95]. The inter-laboratory variability in experimental design, delivery methods, and evaluation criteria further complicates the standardization of reprogramming protocols. Additionally, the properties of the therapeutic cargo, such as plasmids, RNAs, or synthetic chemicals, present unique challenges for delivery, similar to those seen in gene and drug therapy. This section explores potential future directions to overcome these challenges and advance cardiac reprogramming strategies toward clinical use.

### 4.1. Screening New Cardiac Reprogramming Factors in Human Cells

Recent strategies in cardiac reprogramming have primarily focused on well-characterized cardiac-specific transcription factors and microRNAs (Table 1), identified through transcriptomic profiling of heart development. However, high-throughput, scalable screening approaches now offer the potential to de novo identify novel reprogramming factors that may operate independently of traditional developmental mechanisms. Gain-of-function libraries containing hundreds to thousands of transcription factors have been employed to discover previously unexplored candidates, such as Ascl1 and Smad6 [86,96], which had not been implicated in cardiac reprogramming or heart development in earlier studies. Similarly, microRNA mimic libraries and FDA-approved drug libraries have been employed to investigate regulators of cardiac fibroblast biology and heart regeneration [97,98], highlighting their potential to uncover novel small RNA effectors and small molecules relevant to cardiac reprogramming. Conversely, barriers to reprogramming can be elucidated through loss-of-function screenings, such as CRISPR or shRNA-based libraries [44,99]. One limitation of such large-scale screens is the restricted availability of primary CFs. This constraint can be addressed by utilizing human iPSC-derived CFs [100,101], which closely recapitulate key aspects of human CF specification, development, and maturation.

### 4.2. Optimizing Reprogramming Reagents

The rapid advancement of machine learning (ML) and next-generation sequencing (NGS) technologies presents an unprecedented opportunity to accelerate discovery in the field of cardiac reprogramming. By modeling cell fate transitions in silico, ML algorithms can uncover novel transcriptional regulators, enhancer elements, and other molecular players that may function synergistically with established reprogramming factors [96,102]. Leveraging artificial intelligence (AI), particularly deep learning, enables the integration and analysis of high-dimensional, multimodal datasets (including microarrays, single-cell transcriptomics, epigenomics, and proteomics) derived from various cellular reprogramming and differentiation systems [103,104]. These computational frameworks could predict combinatorial reprogramming factor sets with enhanced potential for inducing CM-like phenotypes [105]. Critically, the efficacy of these predicted factors or molecular cocktails can be experimentally validated in MI animal models. The resulting biological outcomes can then be iteratively incorporated back into the computational pipeline, allowing AI models to continuously refine predictive accuracy and optimize reprogramming protocols. This closed-loop, data-driven approach holds great promise for identifying optimal, efficient, and reproducible reprogramming reagents, ultimately advancing the development of clinically viable strategies for cardiac remuscularization and functional heart regeneration following MI.

### 4.3. Engineering New Vesicles for Targeted Gene or Drug Delivery

Owing to their clinical advantages, AAVs have undergone substantial technological refinement to enhance tissue tropism and specificity, increase transduction efficiency, minimize off-target gene delivery, and evade pre-existing humoral immune responses in cardiovascular disease interventions [106]. Future efforts are likely to focus on developing CF-targeted or enriched AAV variants via structure-guided capsid evolution strategies [107], which can be validated in fibroblast lineage-tracing systems. Concurrently, shortened versions of CF-specific enhancers or promoters, engineered to retain both specificity and efficacy, may be re-optimized through deep learning frameworks [108], informed by fibroblast-inclusive chromatin sequencing datasets, and incorporated into AAV constructs. Interestingly, AAV concatemerization may occur via spatial transcriptional crosstalk between tissue-specific enhancers or promoters encoded in separate vectors, enabling the targeted delivery of large genetic cargo [109]. Together, these innovative strategies will broaden the toolkit for designing new CF-specific AAVs and enable precise expression of cardiac reprogramming factors in infarcted myocardium.

Synthetic mRNAs and small molecules have shown promising potential for inducing in vivo reprogramming of CFs into CM-like or cardiovascular cells [48,64,110]. However, when introduced into the myocardial circulatory environment, early delivery methods using naked molecules without protective carriers could suffer from suboptimal stability, limited tissue penetration, inefficient cellular uptake, and significant off-target effects [111]. Furthermore, their broad and non-specific effects on multiple signaling pathways would complicate the assessment of reprogramming outcomes and hinder therapeutic evaluation. Encouragingly, the incorporation of nanocarriers can markedly enhance the delivery efficiency of modified mRNA following intramyocardial injection [112]. Additionally, nanoparticles offer a viable strategy for delivering poorly water-soluble small molecules such as Wnt or TGF-β inhibitors, which are frequently used in cardiac reprogramming, into CFs while preserving their biological activity [113], thereby strengthening the feasibility of in vivo application.

Advances in the design of synthetic nanoparticle delivery systems are opening new opportunities for precision cardiac fibrosis therapy. Conjugations or modifications of these nanoparticles with fibroblast-specific ligands, bioactive peptides (e.g., anti-fibroblast activation protein [anti-FAP]), or fibrosis-associated ECM components can markedly improve their targeting specificity [114,115]. Such molecular engineering not only increases therapeutic payload accumulation in fibrotic cardiac regions but also minimizes off-target effects. As a result, CF-targeted nanoparticles hold strong promise for facilitating the clinical translation of synthetic mRNA and small molecules with anti-fibrotic activity. Beyond fibrosis resolution, these platforms also present an exciting frontier for in vivo cardiac reprogramming, enabling targeted delivery of synthetic mRNA, microRNAs, or small molecules capable of inducing direct CF-to-CM conversion. These possibilities warrant rigorous preclinical evaluation to guide their path toward clinical adoption.

### 4.4. Establishing Stringent Animal Models to Assess Therapeutic Effects

**Figure 2:**
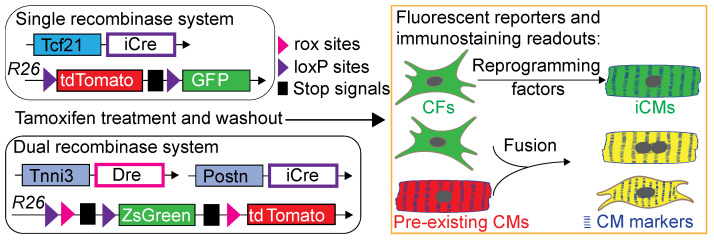
Recent methodological advances for distinguishing reprogrammed cells from fusion-derived cells. Single-lineage [65] or dual-lineage [39] tracing approaches using fluorescent reporters have been implemented to trace iCMs derived from specific CFs. In contrast, cell fusion events are marked by the presence of dual fluorescent signals, allowing for their clear identification.

Fibroblast-lineage tracing models, such as Tcf21-iCre, have been widely utilized to investigate the mechanisms of reprogramming factor activity and to evaluate their therapeutic effects (Table 1). However, given the generally low efficiency of iCM conversion, it is important to consider that the detection of labeled CFs expressing CM markers may be confounded by cell fusion events between pre-existing CMs and CFs [65], particularly under MI conditions. These observations raise similar concerns in the cardiac stem cell-based studies, which have also demonstrated only minimal cardiomyogenic potential [116,117]. Therefore, more stringent lineage-tracing strategies incorporating additional molecular indicators are necessary to rigorously assess the mechanisms and fidelity of cell fate conversion (Figure 2), especially when exploring novel factors for in vivo cardiac reprogramming.

Evaluating cardiac reprogramming therapies in large-animal models is a critical step toward clinical translation. While significant progress has been achieved in rodent models of in vivo cardiac reprogramming, studies in large animals remain limited. Promisingly, fibroblasts from pigs and humans have been reprogrammed into iCMs using cocktails of transcription factors or microRNAs [118]. Interestingly, a microRNA combo has been tested on fibroblasts derived from mice, pigs, dogs, and humans, yielding comparable iCM reprogramming outcomes across species [119]. This suggests that microRNAs may possess superior cross-species reprogramming potential compared to transcription factor-based approaches, which often exhibit species-specific efficacy. These in vitro findings underscore the need for expanded large-animal studies, particularly those leveraging conserved microRNAs. Furthermore, by incorporating nanocarriers with fibroblast-targeting specificity (as discussed above), the delivery of reprogramming factors can be optimized in terms of biodistribution, cellular uptake, retention time, and therapeutic duration. These optimizations should be coupled with assessments of efficacy in large-animal heart failure models to facilitate clinical translation.

## 5. Summary

Collectively, we explore recent advances in the mechanisms underlying in vivo cardiac reprogramming, including the role of fibroblast activation states, the effects of reprogramming factors, and the influence of intercellular communication. Emerging single-cell technologies offer powerful tools for accurately identifying fibroblast phenotypes amenable to reprogramming, as well as for mapping the spatial and temporal dynamics of cellular reprogramming at the tissue level. The integration of ML and AI with high-throughput screening platforms is poised to accelerate the optimization of reprogramming regimens, whether based on cardiac transcription factors or microRNAs. In parallel, the development of fibroblast-specific delivery systems, including AAV-based and non-viral vectors, is essential for translating this strategy into effective anti-fibrotic therapies in preclinical models. Ultimately, progress in this field will require interdisciplinary collaboration across cellular reprogramming, nanomedicine, systems biology, and translational cardiology. With continued effort and innovation, in vivo cardiac reprogramming may evolve into a transformative therapy for heart failure, offering a reparative strategy that goes beyond symptomatic management to true myocardial regeneration.

## Figures and Tables

**Table 1:** Recent advances in gene–drug therapies for in vivo cardiac reprogramming.

Reagents	Animal Model	In Vivo Conversion Efficiency	Function Improvements	Year
Retroviruses (Gata4, Mef2c, Tbx5); Thymosin β4 protein	Postn-Cre;R26-lacZ or Fsp1-Cre;R26-lacZ mice	~12% of retrovirus-infected cells in border/infarct tissue sections (Langendorf CM dissociation or immunohistochemistry)	12 weeks post-MI EF: ~32% vs. ~22% (negative control); 8 weeks post-MI scar size: ~18% vs. ~42%	2012 [19]
Retroviruses (Gata4, Hand2, Mef2c, Tbx5)	Fsp1-Cre;R26-lacZ or Tcf21-iCre;R26-tdT mice	2.5–6.5% of total CMs in border/infarct tissues (Langendorf CM dissociation or immunohistochemistry)	12 weeks post-MI EF: ~57% vs. ~30% (negative control); 4 weeks post-MI scar size: ~20% vs. ~45%	2012 [20]
Retroviruses (Gata4, Mef2c, Tbx5)	αMHC-GFP or nude mice	1–3% of retrovirus-infected cells (immunohistochemistry)	Not defined	2012 [51]
Lentiviruses (Gata4, Mef2c, Tbx5) and adenovirus (VEGF)	Fisher 344 rats	Not defined	7 weeks post-MI EF: 63 ± 2% vs. 48 ± 2% (negative control); 7 weeks post-MI fibrotic area: 12 ± 2% vs. 24 ± 3%	2012 [52]
Lentiviruses (microRNAs-1, 133, 208, and 409)	Fsp1-Cre;R26-tdT mice	~1% of total CMs in infarct tissues (immuno-histochemistry	Not defined	2012 [53]
Lentivirus (polycistronic Gata4, Mef2c, Tbx5) and adenovirus (VEGF)	Fisher 344 rats	Not defined	7 weeks post-MI EF: 48 ± 3% vs. 39 ± 3% (negative control); 7 weeks post-MI fibrotic area: 21 ± 1% vs. 31 ± 6%	2014 [54]
Retrovirus (polycistronic Gata4, Mef2c, Tbx5)	Postn-Cre;R26-lacZ mice	11–36% of lacZ+ cells in border/infarct tissue sections (immunohistochemistry)	8 weeks post-MI EF: ~38% vs. ~18% (negative control); 4 weeks post-MI scar size: ~20% vs. ~40%	2015 [55]
Lentiviruses (microRNAs-1, 133, 208, and 409)	Fsp1-Cre;R26-tdT mice	~12% of total CMs in the peri-infarct tissue sections (immunohistochemistry)	3 months post-MI FS: ~30% vs. ~20% (negative control); 1-month post-MI Fibrosis: ~10% vs. ~25%.	2015 [56]
Retroviruses (Gata4, Mef2c, Tbx5); Small molecules (SB431542, XAV939)	Postn-Cre:R26-YFP mice	150–200 YFP+ iCMs in multiple heart sections (immunohistochemistry)	12 weeks post-MI EF: ~35% vs. ~22% (negative control); 12 weeks post-MI scar size: ~12% vs. ~38%	2017 [57]
Adenoviruses or lentiviruses (Gata4, Mef2c, Tbx5)	Sprague Dawley rats	Not defined	4 weeks post-MI change in EF: echocardiography: 58 ± 3% (Adeno-) or 50 ± 4% (Lent-) vs. 51 ± 2% (negative control); MRI: 60 ± 2% (Adeno-) or 57 ± 3% (Lent-) vs. 56 ± 2%; 4 weeks post-MI fibrotic area: ~6% vs. ~10%	2017 [58]
Sendai viruses (Gata4, Mef2c, and Tbx5)	Tcf21-iCre;R26-tdT mice	~1.5% of tdT+ cells	4 weeks post-MI EF: ~35% vs. ~25% (negative control); 4 weeks post-MI fibrotic area: ~10% vs. ~30%	2018 [59]
AAVs (Gata4, Mef2c, Tbx5, and thymosin β4)	Mice of undefined background	Not defined	Decreased fibrotic area, increased LV wall thickness, increased capillary density, decreased apoptosis, and increased CM markers	2018 [60]
Cationic gold nanoparticles/PEI (Gata4, Mef2c, and Tbx5 plasmids	C57BL/6 J mice	Not defined	Decreased scar or fibrosis area, increased infarct thickness, and increased CM markers	2019 [61]
AAVs (microRNAs-1, 133, 208, and 409)	Fsp1-Cre;R26-tdT mice	~19% of total CMs in peri-infarct region (immunohistochemistry)	Not defined	2020 [62]
Peptide-modified mesoporous silicon nanoparticles (microRNAs-1, 133, 208, and 409)	Fsp1-Cre;R26-tdT mice	~1.5% of tdT+ cells in the infarct area (immunohistochemistry)	4 weeks post-MI EF: ~50% vs. ~28% (negative control); 4 weeks post-MI Scar size in total LV area: ~25% vs. ~60%	2021 [63]
Modified mRNA (Gata4, Mef2c, Tbx5, Hand2) and reprogramming-helper genes (dominant-negative (DN)-TGFβ, DN-Wnt8a, and acid ceramidase)	Tnnt2-iCre;R26-mTmG mice	24% of total non-CMs (mT+ cells) in the infarct area (immunohistochemistry)	4 weeks post-MI EF: 42% vs. 20% (negative control); 4 weeks post-MI fibrotic size: 10% vs. 17%; increased survival rate; increased capillary density	2021 [64]
Sendai Viruses (Gata4, Mef2c, Tbx5)	Tcf21-iCre;R26-mTmG mice	1.0–1.5% of mG+ cells in the infarct myocardium (immunohistochemistry). Fused cells counted for ~0.3%.	Not defined	2021 [65]
Lentivirus (Gata4, Mef2c, Tbx5, Sall4, MyoCD) and small molecules (ruxolitinib and SB431542)	Tnni3-Dre;Postn-iCre;IR1-ZsGreen/tdT or Tnni3-Dre;R26-iCre;IR1-ZsGreen/tdT mice	~28% of Postn-traced cells or ~40% of R26-traced non-CM cells (immunohistochemistry). Fused cells counted for ~4%.	4 weeks post-MI EF: ~34% vs. ~17% (negative control)	2023 [39]
Chemical cocktail (CHIR99021, RepSox, Forskolin, VPA, Parnate, TTNPB, Rolipram)	Fsp1-Cre:R26-tdT or Pdgfrα-DreER;R26-tdT mice	~0.9% of tdT+ cells in the heart sections (immunohistochemistry)	Not defined	2024 [48]
AAVs (Gata4, Hand2, Tbx5, and Mef2c-fused with MYOD transactivation domain)	Postn-iCre:R26-tdT or Tcf21-iCre:R26-tdT mice	~1.8% of tdT+ cells at the border area (immunohistochemistry)	4 weeks post-MI EF: ~32% vs. ~25% (negative control); 4 weeks post-MI fibrotic area: ~20% vs. ~45%	2024 [66]
Retrovirus (PHF7)	Tcf21-iCre;R26-mTmG mice	~2% of mG+ cells in the infarct area (immunohistochemistry) Fused cells counted for ~0.4%.	21 days post-MI EF (echocardiography): ~60% vs. 50% (negative control); 14 to 16 weeks post-MI EF (MRI): ~25% vs. ~10%. 16-week fibrosis: ~52 µm^2^ vs. ~102 µm^2^; increased survival rate	2025 [67]

## Data Availability

No new data were created in this review article.

## Abbreviations

The following abbreviations are used in this manuscript:

CF	Cardiac fibroblast
CM	Cardiomyocyte
iCM	Induced cardiomyocyte
MI	Myocardial infarction
iPSC	induced pluripotent stem cell
ECM	Extracellular matrix
AAV	Adeno-associated virus
MEF	Mouse embryonic fibroblast
EV	Extracellular vesicles
PEI	Polyethylenimine
AI	Artificial intelligence
ML	Machine learning
NGS	Next-generation sequencing
